# A modular toolbox for gRNA–Cas9 genome engineering in plants based on the GoldenBraid standard

**DOI:** 10.1186/s13007-016-0101-2

**Published:** 2016-02-01

**Authors:** Marta Vazquez-Vilar, Joan Miquel Bernabé-Orts, Asun Fernandez-del-Carmen, Pello Ziarsolo, Jose Blanca, Antonio Granell, Diego Orzaez

**Affiliations:** Instituto de Biología Molecular y Celular de Plantas (IBMCP), Consejo Superior de Investigaciones Científicas, Universidad Politécnica de Valencia, Camino de Vera s/n, 46022 Valencia, Spain; Centro de Conservación y Mejora de la Agrodiversidad Valenciana (COMAV), Universidad Politécnica de Valencia, Camino de Vera s/n, 46022 Valencia, Spain

**Keywords:** Plant gene editing, Plant gene activation, Plant gene repression, CRISPR/Cas9, gRNAs, Multigenic assemblies, GoldenBraid, Luciferase/renilla assay

## Abstract

**Background:**

The efficiency, versatility and multiplexing capacity of RNA-guided genome engineering using the CRISPR/Cas9 technology enables a variety of applications in plants, ranging from gene editing to the construction of transcriptional gene circuits, many of which depend on the technical ability to compose and transfer complex synthetic instructions into the plant cell. The engineering principles of standardization and modularity applied to DNA cloning are impacting plant genetic engineering, by increasing multigene assembly efficiency and by fostering the exchange of well-defined physical DNA parts with precise functional information.

**Results:**

Here we describe the adaptation of the RNA-guided Cas9 system to GoldenBraid (GB), a modular DNA construction framework being increasingly used in Plant Synthetic Biology. In this work, the genetic elements required for CRISPRs-based editing and transcriptional regulation were adapted to GB, and a workflow for gRNAs construction was designed and optimized. New software tools specific for CRISPRs assembly were created and incorporated to the public GB resources site.

**Conclusions:**

The functionality and the efficiency of gRNA–Cas9 GB tools were demonstrated in *Nicotiana benthamiana* using transient expression assays both for gene targeted mutations and for transcriptional regulation. The availability of gRNA–Cas9 GB toolbox will facilitate the application of CRISPR/Cas9 technology to plant genome engineering.

**Electronic supplementary material:**

The online version of this article (doi:10.1186/s13007-016-0101-2) contains supplementary material, which is available to authorized users.

## Background

Since its discovery, the clustered regularly interspaced short palindromic repeats (CRISPR)-Cas immune bacterial system has rapidly become a powerful technology for genome editing in many organisms. This system is based on a guide RNA (gRNA) that directs the *Streptococcus pyogenes* Cas9 nuclease to its target site. The application of the RNA-guided Cas9 technology is being widely exploited by the scientific community in cell cultures [1], animals [2, 3] or plants [4, 5].

On the plant field, RNA-guided genome engineering via Cas9 has been employed in diverse approaches, from single and/or multiple gene knock-outs [6–8] to targeted insertions of donor sequences [9] or even targeted transcriptional regulation through the fusion of transcriptional activation or repressor domains to an inactivated Cas9 [10]. A remarkable feature of gRNA–Cas9 is that facilitates targeting multiple sequences simultaneously. While similar technologies such as the ZFNs (zinc finger nucleases) [11] or the TAL effectors [12] require recoding of a new protein for each target sequence, with the gRNA–Cas9 a change of 20 nts in the guide RNA is enough, paving the way for multiplex editing and design of complex regulatory circuits among other engineering possibilities [13].

The direct transfection of Cas9 and guide RNAs into plant protoplasts followed by plant regeneration from single-cell has been shown effective for genome editing in rice and tobacco, however the efficiency remained relatively low, and besides, whole plant regeneration from protoplasts is not currently feasible for many crop species [14]. A successful alternative for plants is the use of Agrobacterium mediated T-DNA transformation, followed by callus induction and organogenic plant regeneration (or floral dip transformation in the case of Arabidopsis). In this case, T-DNA-delivered gRNA–Cas9, besides acting transiently during callus formation, can also integrate in the genome and continue its activity in somatic tissues [4]. To exploit the full potential of the T-DNA strategy it is important to expand the ability to combine different gRNAs together with Cas9 within a single T-DNA, as it has been demonstrated that all-in-one plasmid approaches significantly increase editing efficiency [15].

Modular cloning methods are being increasingly adopted by the plant research community as they greatly facilitate the combinatorial assembly of pre-made DNA elements into multigene constructs [16, 17]. GoldenBraid is a modular cloning standard that makes use of the Type IIS restriction enzyme BsaI for the assembly of basic, so-called “level 0” DNA elements (promoters, coding regions, terminators, etc.) into transcriptional units (TUs), and then incorporates a second enzyme, BsmBI, to build higher level structures using a double-loop iterative strategy [18]. Level 0 parts are flanked by 4 nucleotides overhangs, the sequence of which determines the relative position of each part in the transcriptional unit. To be usable in GB cloning, all level 0 parts need to be previously adapted with the incorporation of flanking BsaI recognition sites, the addition of flanking 4 bp standard barcodes, and the removal of internal BsmBI and BsaI sites. The whole process of adaptation to the standard is often referred to as “domestication”. Once domesticated, GB parts can be efficiently combined to create large multigenic constructs within binary destination plasmids ready to be used in Agrobacterium-mediated plant transformation. A key feature of GB is that all constructs can be reused in new combinations following the same cloning scheme, fostering the exchange of genetic elements. Interestingly, GB part reusability enables the unequivocal association of physical parts with experimental information, as no further modifications (i.e. subcloning, re-assembly or PCR re-amplification) are required to incorporate a GB part into different genetic modules. The GB webpage (https://gbcloning.upv.es/) offers a set of online tools for ‘in silico’ multigenic assemblies and a database for the collection and exchange of GB standard parts [19]. Although Type IIS cloning methods have been employed for multi-gene assemblies with a wide range of applications in several organisms [20, 21], the GB framework is specially designed for plants since the GB destination plasmids are two sets of binary vectors (one based on pGreen and a second one based on pCambia) and all the GB standard parts including promoters and terminators are suitable for plant biotechnology.

The GB cloning strategy is especially suited for the construction of vectors incorporating Cas9 together with multiple guide RNAs in the same T-DNA. Here, we report the implementation of a GB-adapted gRNA–Cas9 toolbox for plants, which includes the domestication of gRNA/Cas9 elements, the definition of a CRISPR cloning workflow and incorporation of new online tools for building CRISPR-based genome engineering constructs in binary vectors.

## Results

### GB-adapted cloning strategy for CRISPR/Cas9 plant constructs

**Fig. 1:**
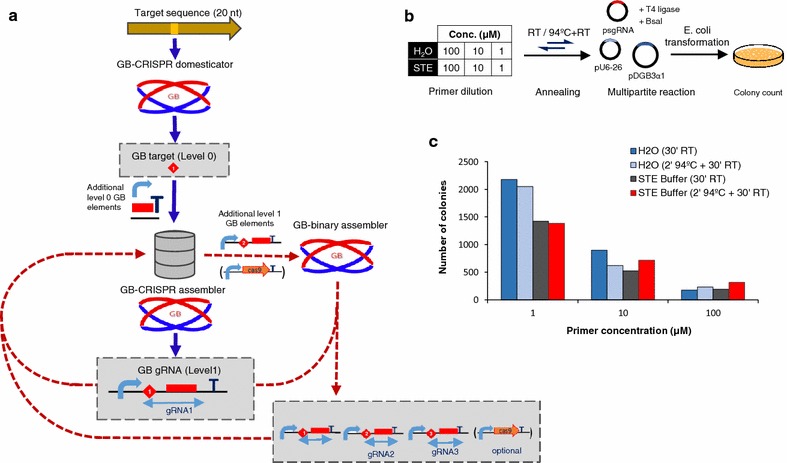
Multiple guide RNAs assembly with GoldenBraid. **a** Software-assisted CRISPR cloning workflow. Targets are adapted to the GoldenBraid standard with the ‘GB-CRISPR domesticator’. Then, these level 0 parts (D/M-Targets) are combined with other standard GBparts with the ‘GB-CRISPR assembler’ to create the guide RNA expression cassettes, which can be combined between them and/or with a Cas9 transcriptional unit with the ‘GB-binary assembler’. **b** Optimization of GB-CRISPR multipartite reactions. Forward and reverse primers were diluted to different concentrations with different solvents; they were mixed and twelve independent multipartite reactions were set up. After transformation into *E. coli*, the number of colonies was estimated. **c** Number of colonies obtained on the twelve independent guide RNA multipartite assembly reactions

To facilitate the assembly of CRISPR/Cas9 constructs and the delivery of multiple guide RNAs in the same T-DNA, we designed the CRISPR cloning workflow depicted on Fig. 1a. As a first step, twenty nucleotides sequences designed against a specific genomic target can be incorporated to the GoldenBraid scheme using the ‘GB CRISPR domesticator’ tool available at https://gbcloning.upv.es/do/crispr/. This tool generates a new target-specific GB element (D-Target/M-Target, syntax structure B3c–B4–B5c or B3c–B4–B5d), which can be used immediately or stored in the database for future assemblies. The D/M-Target comprises two partially complementary oligonucleotides yielding a double-stranded DNA fragment flanked by four nucleotides overhangs. In a next step, the D/M-Target is combined with a PolIII promoter (currently, Arabidopsis U6-26 and U6-1 and rice U3 promoters are available in the GB collection) and with the scaffold RNA in a cyclic digestion/ligation Golden Gate reaction [22] to build the complete gRNA expression cassette. This step is assisted by the ‘CRISPR Assembler’ tool available at https://gbcloning.upv.es/tools/crisprsassembler.

The conditions for gRNA assembly were optimized by checking three key parameters, namely primer concentration, primer dilution buffer and annealing conditions in a total of 12 combinations. The resulting assemblies were then transformed into *E. coli* and the efficiency assessed by the number of colonies obtained (Fig. 1b, c). Two colonies of each of the 12 assembly reactions were selected for restriction analysis resulting in a 100 % of positive clones (see Additional File 1: Figure S1). Primer dilution was found the main factor affecting reaction efficiency, with best results obtained at low primer concentrations. Only minor effects were observed associated to buffer or denaturing condition (Fig. 1b, c). Accordingly, recommended conditions for CRISPR assembly in multipartite GB reactions were set at 1 μM primer concentration in water with a 30 min annealing step performed at room temperature.

Following the GB workflow, every gRNA expression cassette assembled in GB compatible vectors can be combined with each other and/or with a Cas9-encoding transcriptional unit (Fig. 1a) with the ‘GB Binary Assembler’ web tool (https://gbcloning.upv.es/do/bipartite/). GB binary reactions were highly efficient as previously described Sarrion-Perdigones et al. [23] and accurate since white colonies analyzed resulted in 100 % correct assemblies in most cases (see Additional file 1: Figure S1; Additional file 2: Table S3). The current GB-adapted gRNA–Cas9 toolbox incorporates seven different Cas9-encoding TUs which have been designed for gene editing, gene activation and gene repression projects. All Cas9 TUs described in this paper were created by combining only protein-coding GBparts, leaving constitutive plant expression elements invariant. The assembly of inducible and/or tissue-specific expression of Cas9 is also possible using other standard parts from the collection.

### Transient expression of GB-adapted Cas9 TUs provides efficient targeted mutagenesis in *N. benthamiana* leaves

**Fig. 2:**
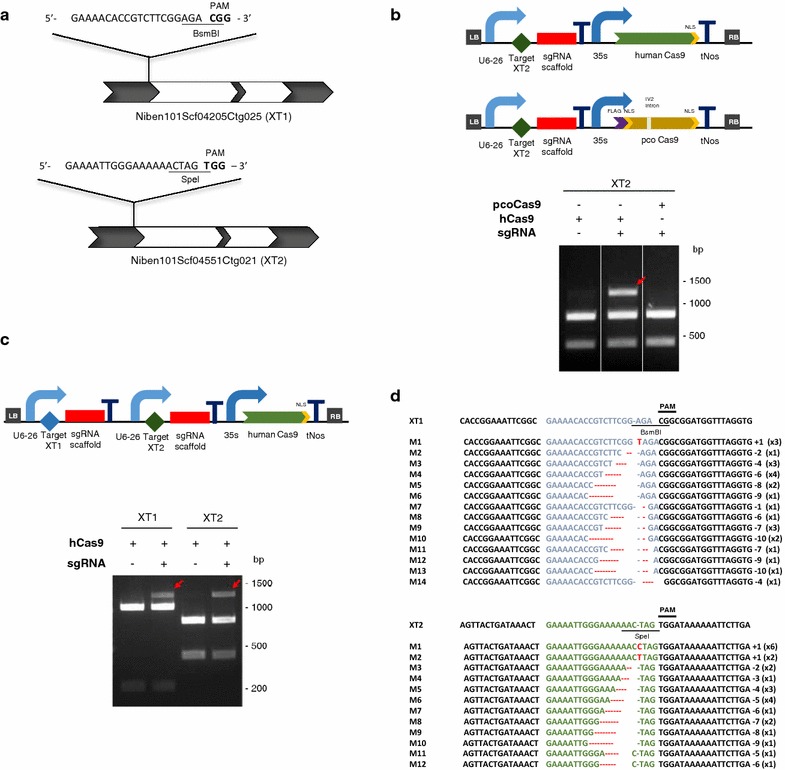
Targeted mutagenesis using the CRISPR/Cas9 system in transient expression in *N. benthamiana.* **a** Schematic representation of the structure of Niben101Scf04205Ctg025 (XT1) and Niben101Scf04551Ctg021 (XT2) (exons in *grey*, introns in *white*) with the sequences of the target sites. Diagnostic restriction sites are underlined and the PAM sequence is shown in *bold*. **b** Comparison of the mutation efficiency of hCas9 and pcoCas9 targeting the XT2. *Red arrow* shows SpeI resistant PCR fragments only visible on the gRNA and hCas9 combination. **c** PCR/RE assay to detect simultaneous targeted mutations on XT1 and XT2. *Red arrows* show BsmBI and SpeI resistant PCR fragments amplified from *N. benthamiana* genomic DNA. **d** Alignment of XT1 and XT2 sequences obtained from different clones of uncleaved bands (see **c**). XT1 target site appears in blue and XT2 target site in *green*. *Red letters* and *dashes indicate* insertions and deletions respectively

To experimentally validate the different GB modules for gRNA–Cas9-mediated gene mutation, we tested them in *N. benthamiana* by targeting the endogenous xylosyltransferase (XT) gene. A BLAST search on the *N. benthamiana* genome with the GenBank accession ABU48858, resulted in scaffolds Niben101Scf04205Ctg025 and Niben101Scf04551Ctg021 corresponding to predicted cDNAs Niben101Scf04205g03008 (XT1) and Niben101Scf04551g02001 (XT2) respectively. We decided to target the two of them using a specific guide RNA for each one. The 20-bp target sequences for each guide RNAs were designed with the CRIPSR-P online tool [24], imposing the requirement for a G at the 5′ end of the sequence and minimizing off-targeting. An extra criterion for selection was the presence of a restriction site overlapping the Cas9 cleavage site to facilitate the detection of the mutations. The selected targets are depicted on Fig. 2a.

GB-based gene targeting constructs carrying human-optimized (h) [25] and plant-optimized (pco) [26] Cas9 variants directed to the single target of XT2 were transferred to Agrobacterium and infiltrated into *N. benthamiana* leaves. To test the mutation efficiency, genomic DNA was extracted from leaves, the targeted region amplified by PCR and the presence of mutated fragments estimated based on the elimination of the internal SpeI restriction enzyme (RE) site. The mutation efficiency for the hCas9 was estimated as 11 % based on the intensity of the undigested band (Fig. 2b Lanes 2 and 3) relative to the undigested DNA present on the negative control (Fig. 2b Lane 1). For pcoCas9 mutation efficiency was below detection levels as it was not possible to visualize the undigested band on the agarose gel.

According to these results we assembled both gRNAs targeting XT1 and XT2 together with the hCas9 TU in a single T-DNA and transiently expressed them in *N. benthamiana* leaves. hCas9-induced mutations were detected as above with the restriction enzyme site loss method using BsmBI for XT1 and SpeI for XT2 (Fig. 2c). The gRNA-guided Cas9 activity resulted in part of the DNA being resistant to RE digestion (see undigested band in Lanes 2 and 4) that was not detected when only hCas9 was expressed (Lanes 1 and 3). To corroborate the presence of mutations on the undigested PCR products, the undigested amplicons were cloned and individual clones were sequenced. The most prevalent mutations observed for XT1 were deletions of less than 10 nucleotides, while for XT2 a 32 % of the mutated clones had single nucleotide insertions (C or T) (Fig. 2d). Mutation rates of 17 % (XT1) and of 14.5 % (XT2) were observed for the new construct. Since 29 % (XT1) and 32 % (XT2) of the clones showed the wild type sequence, we included this correction factor to obtain a more accurate estimation of the mutation rate. As result, we obtained a mutation rate of 12.1 % for XT1 and a mutation rate of 9.9 % for XT2, consistent with the 11 % obtained for the same target when a single gRNA was used. The differences in the mutation efficiencies observed in both targets could be due to a GC content of 30 % for target XT2 in contrast to a 50 % GC content of target XT1.

### GB-adapted dCas9 variants modulate transcriptional activity *in N. benthamiana* transient assays

The modularity of GoldenBraid assembly facilitates the design of Cas9 variants with novel functions as e.g. transcriptional activators, repressors, chromatin remodeling factors, etc., by incorporating additional coding modules as translational fusions to an inactive (dead) version of Cas9 (dCas9). To validate this option we built and tested a number of GB-based transcriptional regulators which were targeted to a nopaline synthase promoter (pNOS) fused to a luciferase reporter.

**Fig. 3:**
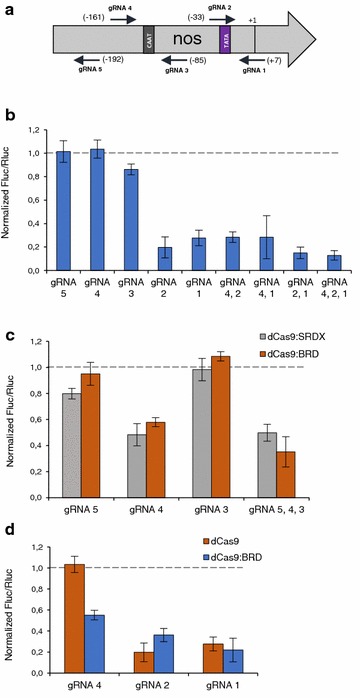
Transcriptional repression of the nopaline synthase promoter (pNOS) with different variants of the dead Cas9. **a** Schematic representation of the gRNA target positions on the pNOS. The gRNAs were selected in both sense and antisense strands. In parenthesis the 5′ position of each gRNA according to the pNOS transcription start site. **b** Comparison of the repression rates mediated by the different gRNAs combinations targeting the pNOS in combination with the dCas9. **c** Repression rates of the dCas9:BRD and dCas9:SRDX in combination with gRNAs targeting different positions upstream the pNOS TATA-box. **d** Influence of the presence of the BRD domain fused to the dCas9 on the repression levels induced by gRNAs 1, 2 and 4. All values were normalized to the Fluc/Rluc ratios of a reference sample set as 1. *Bars* represent average values of three samples ± SD

Making use of level 0 standard genetic parts, we assembled five different transcriptional units (TUs) expressing either the dCas9 (D10A H840A) alone or C-terminus chimeric versions of it fused either to an activator (VP64 or EDLL) or a repressor (SRDX and BRD) (Additional file 1: Figure S2). These five chimeric transcriptional regulators were tested in combination with five gRNAs directed against different regions of pNOS on both sense and antisense strands (Fig. 3a). Changes in the transcriptional activity in these construct were estimated with the luciferase/renilla system using a reporter construct (REP) that included the firefly luciferase (Fluc) driven by the pNOS and the renilla luciferase (Rluc) driven by the 35S promoter as an internal reference. Transient co-transformations of REP with Cas9 and gRNA constructs were performed in order to test the ability of GB-built dCas9 chimeras to modulate transcription.

Since previous studies reported that dCas9 itself could act as a transcriptional repressor [27], we first tested the repressor activity of the non-chimeric dCas9 TU. All five gRNAs targeting pNOS induced variable repression rates depending on their position (Fig. 3b). The Fluc/Rluc ratios decreased as the position of the gRNA gets closer to the Transcription Start Site (TSS) whereas no repression was detected neither for gRNA4 (positions −161 to −142) nor for gRNA5 (positions −211 to −192). Co-expression of the two most effective gRNAs, gRNA 1 and 2, showed a nearly additive effect. However, the addition of a further gRNA, such as gRNA4, to one or both of them did not change the repression level.

Next, the dCas9 fusions to the BRD and the SRDX repressor domains were tested in combination with gRNAs 3, 4 and 5, all three designed to bind upstream the TATA-box. Figure 3c shows that only gRNA4, the gRNA designed on the sense strand, was capable of producing a significant repression on the transcriptional activity. A slight decrease in the Fluc/Rluc ratio was observed when gRNA4 was combined with the two additional gRNAs. The repression levels found with the dCas9:BRD and dCas9:SRDX were similar (Fig. 3c).

To determine whether the presence of the repressor domain modified the effect of the dCas9 itself, we compared the transcriptional activity obtained for the gRNAs 1, 2 and 4 in presence of the dCas9 with the ones obtained with the dCas9:BRD (Fig. 3d). While in the case of the gRNA4 only dCas9:BRD had an effect on the reduction of the transcriptional activity, for the gRNAs overlapping the TATA-box and the TSS, both dCas9 and dCas9:BRD achieved similar repression levels.

**Fig. 4:**
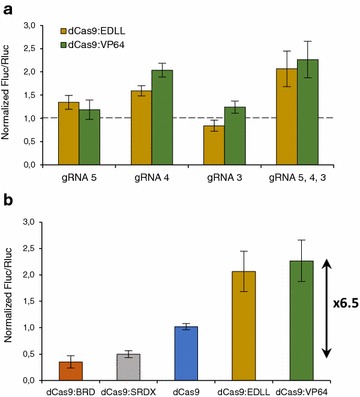
Transcriptional activation and modulation of the nopaline synthase promoter (pNOS). **a** Fluc/Rluc ratios obtained with dCas9:VP64 and dCas9:EDLL in combination with gRNAs 3, 4 and 5. **b** Comparison of the Fluc/Rluc ratios obtained for gRNAs 3, 4 and 5 in combination with the different dCas9 variants reported on this work. All values were normalized to the Fluc/Rluc ratios of the reference sample set as 1. *Bars* represent average values of three samples ± SD

Next, we decided to test whether the dCas9 fused to an activator domain could increase the transcriptional activity on the same reporter construct. The results showed that dCas9:VP64 and dCas9:EDLL raised the reporter levels in combination with gRNA4, while in combination with gRNA5 only a small induction rate was detected and no induction was observed with gRNA3, corroborating the functionality observed for the same gRNAs with dCas9:SRDX and dCas9:BRD (Fig. 4a). Using both the dCas9:VP64 and the dCas9:EDLL variants in combination with 3× multiplexed gRNAs (gRNA 3, 4 and 5), the pNOS transcriptional activity was doubled.

These results demonstrated that it is possible to modulate the transcriptional activity driven by the pNOS using one or more gRNAs in combination with different chimeric versions of the dCas9. The maximum induction rate, calculated with the values of the best reported repression and activation Fluc/Rluc ratios, was 6.5× (Fig. 4b).

### Second-dimension multiplexing using GoldenBraid

**Fig. 5:**
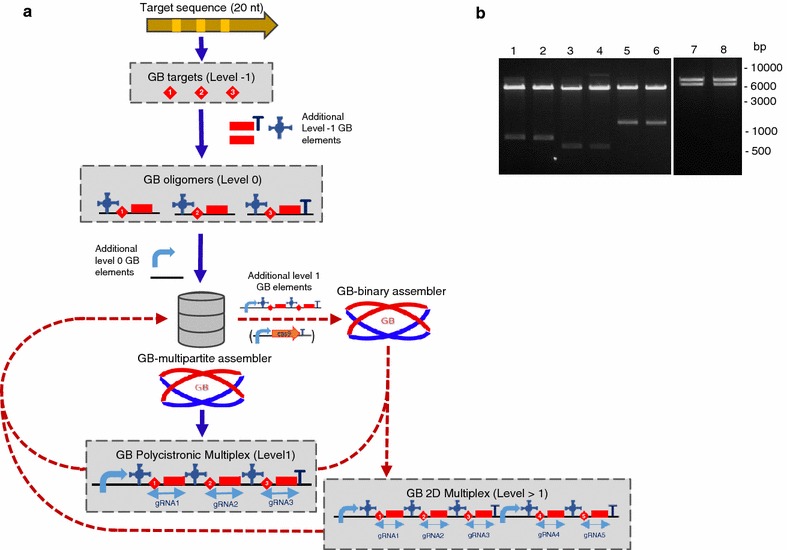
Second dimension Multiplexing with Goldenbraid. **a** Pipeline of the 2D multiplexing strategy. Targets are designed as level 0 structures and combined with standard level −1 parts to create individual oligomers that are combined in level 1 polycistronic tRNA–gRNA structures. The binary combination of two polycistrons incorporates a 2D multiplexing step on the CRISPR cloning workflow. **b** Restriction analysis of two clones of level 1 polycistronic structures targeting fucosyl (*Lanes 1* and *2*; *EcoRI* expected bands: 6345-796) and xylosyltransferases (*Lanes 3* and *4*; *HindIII* expected bands: 6345-623), two clones of a level 2 construct derived from their binary assembly (*Lanes 5* and *6*; *BamHI* expected bands: 6674-1401) and two clones of its assembly with the hCas9 (*Lanes 7* and *8*; *BsmBI* expected bands: 7215-6367)

To further increase the gRNA multiplexing capacity we decided to incorporate a polycistronic strategy to the GB pipeline. This strategy, which has been validated in rice [28], allows the simultaneous expression in a single transcript of multiple gRNAs, which are later processed by the endogenous tRNA ribonucleases P and Z to produce the individual gRNAs. To adapt the general GB cloning system to the polycistronic strategy we incorporated single tRNA–gRNA oligomers as level 0 GBparts, which are then multipartitely assembled on level 1 to create polycistronic tRNA–gRNAs (Fig. 5a). To avoid using PCR reactions during the construction of each tRNA–gRNA oligomer, we designed new level −1 plasmids containing both the tRNA and the gRNA flanked by BsmBI restriction sites. The BsmBI assembly of level −1 plasmids with the D-target primers heteroduplex results in level 0 GB-oligomers. In turn, these level 0 elements are combined together with the level 0 PolIII promoter to create a level 1 polycistronic tRNA–gRNA in a software-assisted step available at https://gbcloning.upv.es/do/multipartite/free/. We validated the assembly efficiency of the 2-D multiplexing schema by assembling a level 2 construct targeting simultaneously *N. benthamiana* fucosyl and xylosyltransferase genes. As the two gRNAs targeting XTs have been previously tested in this work, we used the same targets (Additional file 2: Table S2) for the assembly of a polycistronic tRNA–gRNA combining two GBoligomers. Since the number of genes encoding fucosyltransferases in the *N. benthamiana* genome is very high, we decided in this example to target only five of them using a combination of three gRNAs (Additional file 2: Table S2), one of them targeting three genes and the remaining two gRNAs targeting a single gene. After assembling firstly all five level 0 oligomers and subsequently the two level 1 polycistronic structures, they were combined together in a GB binary reaction (Fig. 5b) to generate a single binary plasmid containing all five gRNAs targeting a total of seven genes encoding fucosyl and xylosyltransferases. All the assembly steps resulted in 100 % accuracy rates (at least 4 white colonies analysed in each step) demonstrating the efficiency of the proposed scheme for 2D multiplexing. The whole process took just nine working days, and in three extra days the Cas9 was added to the assembly.

## Discussion

The adoption of standard rules and modular design has promoted the expansion of many engineering disciplines from mechanics to electronics and is likely to have an impact in genome engineering as well. Modular cloning methods based on TypeIIS restriction enzymes such as Golden Gate [22], MoClo [29] and GoldenBraid [23], greatly facilitate the construction of large multigene assemblies enabling the concurrent delivery of multiple pieces of genetic information into the cell. Moreover, Type IIS cloning systems are especially well suited for the definition of standard assembly rules. Very recently, a common DNA assembly syntax for TypeIIS cloning has been agreed by 26 different Plant laboratories and research groups worldwide, constituting one of the first examples of a Bioengineering Standard adopted by the Scientific Community [16]. We have introduced the necessary modifications in GB to make the gRNA–Cas9 toolbox fully compliant with the new standard.

The first step towards GB adaptation for gene targeted mutation consisted in the design of a GB-compatible assembly scheme that facilitates both gRNA multiplexing and Cas9 modification. We decided to build both gRNAs and Cas9 transcriptional units as level 1 structures to maximize their exchangeability while preserving the combinatorial potential. In the GB system, level 1 constructs grow only binarely, which poses a certain limitation in terms of cloning speed. Other systems growing multipartitely using Golden Gate assembly have been proposed for mammalian and plant systems, however this is at the cost of flexibility and reusability of the constructs [30–32]. Conversely, level 1 GB constructs are exchangeable, offering the possibility to reuse efficient gRNA constructs in new editing or regulatory combinations. Furthermore, this initial decision proved to be most adequate with the incorporation of polycistronic tRNA–gRNA constructs at level 1, which provides a new combinatorial dimension for multiplexing, and makes possible to hierarchically combine gRNAs using different assembly levels. Hence, in our 2D editing example we grouped homologous functions (either xylosyl or fucosyltransferases) in level 1, and later combined them in level 2 in a binary assembly step. Similarly hierarchical assembly approaches can be used to build increasingly complex gRNA–Cas9-based transcriptional regulatory circuits in few days.

The assembly and functional validation of several gRNA–Cas9 constructs provides evidence of the efficiency of the process and the functionality of the elements that were incorporated to the GB toolkit. GB is based on Golden Gate typeIIS cloning which is an extremely efficient multipartite assembly method when parts are conveniently cloned within an entry plasmid. Whether the same high efficiency is maintained when one of the parts is made of two partially overlapping 23–25 mer oligonucleotides encoding the target sequence remained to be tested. Counterintuitively, the efficiency of the reaction was shown to be significantly higher when low concentrations of oligonucleotides (nM range) were employed in the reaction mix. Also, it is worth to notice that in the proposed GB gRNA building scheme, the only variable input specific for each new construct are the two 25 mer oligonucleotides; all the remaining building elements are invariant and stored in the GB collection, a feature that significantly reduces gene synthesis costs for building gRNA–Cas9 constructs for plants.

The first functional characterization of the new GB targeted mutagenesis tools was the quantification of Cas9 nuclease activity in a *N. benthamiana* transient expression method [26, 33]. As shown, efficiencies up to 12 % were observed using a human codon optimized Cas9 (hCas9) directed against two independent targets. In our hands hCas9 performed better than plant-optimized pcoCas9 in *N. benthamiana* transient assays, although it remains to be seen if the same differences are observed in other experimental systems. The mutation rate observed here with the hCas9 is consistent with those described when hCas9 and gRNAs were assembled in the same T-DNA [34] and much higher than the rates obtained by [34] and [33] when the same were co-delivered in different plasmids by in *trans* co-transformation. The reported efficiency for the plant-optimized pcoCas9 when co-expressed with the gRNA on the same vector was substantially lower (4.8 %) [26]. Therefore it is possible that our detection system based on the presence of an undigested band was not sensitive enough to detect this mutation rate.

The ability of GB-adapted gRNA/Cas9 elements to conduct RNA-guided transcriptional regulation was assessed by using the pNOS fused to luciferase as a reporter system. We observed that, by directing a nuclease-inactivated Cas9 to promoter regions around the transcription origin of the reporter gene, expression levels were severely reduced. These results were in line with previous reports showing an intrinsic repressor activity of a dCas9 without further modifications [10, 27]; however in our experimental conditions dCas9 intrinsic repression was almost completely abolished when paired to gRNAs targeting distal regions upstream of the −100 position. In the same upstream regions, however, the translational fusion of dCas9 with specific transcription modulating protein domains efficiently conducted the downregulation (BRD, SRDX) or upregulation (VP64, EDLL) of the reporter activity respectively. It was also observed that, by targeting several gRNAs towards the same promoter, the activation/repression effect was increased, highlighting the convenience of multiplex targeting to achieve efficient transcriptional regulation. Altogether, the range of transcriptional activities that we were able to modulate using current GB gRNA–Cas9 tools was relatively modest, approximately seven times from the strongest repressor to the strongest activator. Further optimization of the system (e.g. improved fusion linkers, optimization of fusion sites, etc.) will be necessary to increase this efficiency. Nevertheless it should be noticed that, given that in the *N. benthamiana* agroinfiltration system several T-DNA copies of the reporter gene are co-delivered simultaneously in each cell there is probably a high demand for dCas9 fusions to achieve substantial activation/repression. In future experiments the quantification of the effect of dCas9 fusions on single copy genes stably integrated in the plant genome will be investigated.

Very recently, new gRNA–Cas9 toolkits for targeted mutagenesis or transcriptional regulation have been reported including animal [35, 36] and plant-dedicated [31, 32, 37] systems, although none of them involve a standardized strategy. Interestingly, the toolbox reported by Lowder et al. incorporates gRNA–Cas9 elements for targeted mutagenesis and transcriptional regulation using a combination of type IIs and gateway recombination for multiplex assembly. In comparison, the GB toolbox showed here present a number of distinctive features. First, the GB toolbox includes a number of software tools that generate standardised protocols in each gRNA–Cas9 assembly step. The implementation of assembly software tools not only serves to facilitate construct-making for non-trained users, but most importantly, it turns GB into a self-contained, fully traceable assembly system, where all elements generated with GB software tools, now including also gRNA/Cas9 elements, are perfectly catalogued and their genealogy documented. Second, the modularity of GB facilitates combinatorial arrangements as e.g. between pre-set gRNA arrays and different Cas9 versions and enables the exchange of pre-made combinations. Finally, the GB cloning loop enables endless assembly of both monocistronic and polycistronic tRNA–gRNA expression cassettes, enhancing the multiplexing capacity of the system.

## Conclusions

A modular gRNA–Cas9 toolbox conforming to the GoldenBraid standard for Plant Synthetic Biology was developed and functionally validated. The GB-gRNA/Cas9 toolbox, comprising an adapted cloning pipeline, domesticated gRNA/Cas9 elements and a dedicated software tool, was shown to facilitate all-in-one-T-DNA cloning and gRNA multiplexing. The GB-adapted gRNA/Cas9 elements combined among them and/or with other GB elements were shown effective in targeting reporter genes for mutagenesis, transcriptional activation and transcriptional repression in *N. benthamiana* transient assays. The GB adaptation enhances CRISPRs/Cas9 technology with traceability, exchangeability and improved combinatorial and multiplexing capacity.

## Methods

### GBparts construction

GBparts used in this work were created following the domestication strategy described in [18]. For parts GB0575, GB1001 and GB1079, PCR amplifications with the primers obtained at https://gbcloning.upv.es/do/domestication/were performed using the Phusion High-Fidelity DNA polymerase (Thermo Scientific). For level 0 parts GB0273, GB0645, GB1175, GB1185, GB1186, GB1187 and for level −1 parts GB1205, GB1206, GB1207 double-stranded DNA was synthesized using IDT gBlocks^®^ Gene Fragments. GB1041 was amplified from GB0575 to incorporate the D10A and H840A mutations. For level 0 parts, 40 ng of the PCR products or gBlocks^®^ were cloned into the pUPD with a *BsmBI* restriction–ligation reaction. Level −1 parts were cloned into the pVD1 (GB0101) with a *BsaI* restriction–ligation reaction following the same protocol. A list of the level −1 and level 0 parts is provided in the Additional file 2: Table S3; their nucleotide sequences can be searched at https://gbcloning.upv.es/search/features/with their corresponding ID numbers. All level −1 and level 0 GB parts were validated by restriction enzyme (RE) analysis and confirmed by sequencing.

### Guide RNA assembly on level 0 and level 1

Assembly optimization reactions were performed as follows: primers gRNA_XT2_F/gRNA_XT2_R were resuspended in water and STE buffer (10 mM Tris pH 8.0, 50 mM NaCl, 1 mM EDTA) to final concentrations of 100, 10 and 1 µM. Equal volumes of forward and reverse primers were mixed. The mixture was split into two different tubes and one of them was incubated at 94 °C for 2 min prior to a 30 min incubation at room temperature while the other was directly incubated at room temperature for 30 min. The BsaI restriction–ligation reactions were set up in 10 µl with 1 µl of primers mix, 75 ng of GB1001 (U626 promoter), 75 ng of GB0645 (scaffold RNA) and 75 ng of pDGB3α1 destination vector. One microliter of the reaction was transformed into *E. coli* TOP10 electrocompetent cells and the number of white colonies growing on agar plates counted.

The selected conditions for the gRNA assemblies were dilution in water, incubation at room temperature for 30 min and set the restriction–ligation reaction with a final primer concentration of 0.1 µM. For gRNA assemblies on level 1, two complementary primers designed at http://www.gbcloning.upv.es/do/crispr/and listed on Additional file 2: Table S2, were included in a *BsaI* restriction–ligation reaction following the selected conditions. For the assembly of guide RNAs on level 0, the primers listed on Additional file 2: Table S2 were included in a *BsmBI* restriction–ligation reaction following the selected conditions together with the pUPD2 and 75 ng of the corresponding level −1 tRNA-scaffold plasmid depending on the desired position of each target on the level 1 assembly. All level 1 gRNA constructs were validated by RE-analysis, analyzed by sequencing and confirmed correct.

### Cloning in α and Ω-level destination vectors

Multipartite BsaI restriction–ligation reactions from level 0 parts and binary *BsaI* or *BsmBI* restriction–ligation reactions were performed as described in [18] to obtain all the level ≥1 assemblies. A list with all the TUs and modules used in this work is provided on the Additional file 2: Table S3. All level ≥1 were validated by restriction enzyme (RE) analysis. Furthermore, partial sequencing was carried out to check part’s boundaries. The sequences of all level ≥1 constructs can be found entering their IDs (displayed at Additional file 2: Table S3) at https://gbcloning.upv.es/search/features/.

### *Nicotiana benthamiana* agroinfiltration

For transient expression, plasmids were transferred to *Agrobacterium tumefaciens* strain GV3101 by electroporation. *N. benthamiana* plants were grown for 5 to 6 weeks before agroinfiltration in a growing chamber compliant with European legislation. Growing conditions were 24 °C (light)/20 °C (darkness) with a 16-h-light/8-h-dark photoperiod. Agroinfiltration was carried out with overnight-grown bacterial cultures. The cultures were pelleted and resuspended on agroinfiltration solution (10 mM MES, pH 5.6, 10 mM MgCl_2_, and 200 μM acetosyringone) to an optical density of 0.2 at 600 nm. After incubation for 2 h at room temperature on a horizontal rolling mixer, the bacterial suspensions were mixed in equal volumes. The silencing suppressor P19 was included in all the assays; in the same T-DNA for the transcriptional regulation experiments and co-delivered in an independent T-DNA for the targeted mutagenesis assays. Agroinfiltrations were carried out through the abaxial surface of the three youngest leaves of each plant with a 1 ml needle-free syringe.

### Genomic DNA extraction and PCR/restriction enzyme assay

Samples for genomic DNA extraction were collected from 5 days post infiltrated leaves. For genomic DNA extraction, 50 mg of tissue powder coming from a pool of three leaves were ground in 500 µl of DNA extraction buffer (200 mM TrisHCl-pH 7.5, 250 mM NaCl, 25 mM EDTA, 0.5 % SDS). The plant extract was mixed gently and it was spin at 14,000×g for 3 min. The supernatant was transferred to a new tube and an equal volume of isopropanol was added for DNA precipitation. The supernatant was removed after centrifugation (5 min at 14,000×g) and the DNA was washed twice with 70 % ethanol. The pellet was dried for half an hour and it was dissolved with 100 µl of elution buffer (10 mM TrisHCl-pH 8, 1 mM EDTA).

DNA amplicons covering the XT1 and XT2 target sites were obtained by PCR of genomic DNA using the Phusion High-Fidelity DNA polymerase (Thermo Scientific) and two pairs of gene specific primers: XT1_F/XT1_R for XT1 and XT2_F/XT2 _R for XT2 (Additional file 2: Table S1). The resulting PCR products were purified with the QIAquick PCR purification kit (QIAGEN) following the manufacturer’s protocol and restriction reactions were set up with 500 ng of purified DNA and the corresponding restriction enzyme; BsmBI (Fermentas) for XT1 and SpeI (Fermentas) for XT2. Band intensities were estimated using the ‘Benchling Gels’ (https://benchling.com) tool.

### Gel band purification and *BsaI*-cloning

PCR products resistant to *BsmBI* and *SpeI* digestion were purified from a 1 % agarose gel with the QIAEX II Gel Extraction Kit following the manufacturer’s protocol. For sequence analysis, the purified PCR products were subsequently amplified with XT12BsaI_F/XT12BsaI_R primers (Additional file 2: Table S1) to incorporate BsaI sites for improving cloning efficiency. Finally, they were cloned into the pDGB3α1 with a *BsaI* restriction–ligation reaction and individual clones were sequenced.

### Luciferase/Renilla activity determination

Samples of leaves coinfiltrated with the REP (GB1116), different activator/repressor TUs (GB1172 and GB1188 to GB1191) and the independent or combined gRNAs targeting the pNOS were collected at 4 days post infiltration. For the determination of the luciferase/renilla activity one disc per leaf (d = 0.8 cm, approximately 18–19 mg) was excised, homogenized and extracted with 150 µl of ‘Passive Lysis Buffer’, followed by 15 min of centrifugation (14,000×g) at 4 °C. Then, the supernatant was diluted 2:3 in Passive Lysis Buffer resulting in the working plant extract. Fluc and Rluc activities were determined following the Dual-Glo^®^ Luciferase Assay System (Promega) manufacturer’s protocol with minor modifications: 10 µl of working plant extract, 40 µl of LARII and 40 µl of Stop&Glo Reagent were used. Measurements were made using a GloMax 96 Microplate Luminometer (Promega) with a 2-s delay and a 10-s measurement. Fluc/Rluc ratios were determined as the mean value of three samples coming from three independent agroinfiltrated leaves of the same plant and were normalized to the Fluc/Rluc ratio obtained for a reference sample including the REP (GB1116) co-infiltrated with an unrelated gRNA (GB1221) and the corresponding activator/repressor TU.

## Additional files

10.1186/s13007-016-0101-2 Cloning efficiency of representative Level ≥1 GBelements. **Figure S2.** Schema of the dead Cas9 transcriptional units tested on the repression and activation experiments.
10.1186/s13007-016-0101-2 Primers used for the amplification of the *N. benthamiana* xylosyltransferases XT1 (Niben101Scf04205Ctg025) and XT2 (Niben101Scf04551Ctg021) regions. **Table S2.** List of forward and reverse primers used to construct the targets. **Table S3.** List of GBelements generated in this work.

## Abbreviations

pNOS: nopaline synthase promoter
gRNA: guideRNA
GB: GoldenBraid
TU: transcriptional unit
XT: xylosyltransferase
Fluc: firefly luciferase
Rluc: renilla luciferase

## References

[1] Ran FA, Hsu PD, Wright J, Agarwala V, Scott DA, Zhang F (2013). Genome engineering using the CRISPR-Cas9 system. Nat Protoc.

[2] Yang X (2015). Applications of CRISPR-Cas9 mediated genome engineering. Mil Med Res.

[3] Wang H, Yang H, Shivalila CS, Dawlaty MM, Cheng AW, Zhang F (2013). One-step generation of mice carrying mutations in multiple genes by CRISPR/Cas-mediated genome engineering. Cell.

[4] Bortesi L, Fischer R (2015). The CRISPR/Cas9 system for plant genome editing and beyond. Biotechnol Adv.

[5] Belhaj K, Chaparro-Garcia A, Kamoun S, Patron NJ, Nekrasov V (2015). Editing plant genomes with CRISPR/Cas9. Curr Opin Biotechnol.

[6] Shan Q, Wang Y, Li J, Zhang Y, Chen K, Liang Z (2013). Targeted genome modification of crop plants using a CRISPR-Cas system. Nat Biotechnol.

[7] Gao J, Wang G, Ma S, Xie X, Wu X, Zhang X (2015). CRISPR/Cas9-mediated targeted mutagenesis in *Nicotiana tabacum*. Plant Mol Biol.

[8] Fauser F, Schiml S, Puchta H (2014). Both CRISPR/Cas-based nucleases and nickases can be used efficiently for genome engineering in *Arabidopsis thaliana*. Plant J..

[9] Schiml S, Fauser F, Puchta H (2014). The CRISPR/Cas system can be used as nuclease for in planta gene targeting and as paired nickases for directed mutagenesis in Arabidopsis resulting in heritable progeny. Plant J.

[10] Piatek A, Ali Z, Baazim H, Li L, Abulfaraj A, Al-Shareef S (2015). RNA-guided transcriptional regulation in planta via synthetic dCas9-based transcription factors. Plant Biotechnol J.

[11] Beerli RR, Barbas CF (2002). Engineering polydactyl zinc-finger transcription factors. Nat Biotechnol.

[12] Bogdanove AJ, Voytas DF (2011). TAL effectors: customizable proteins for DNA targeting. Science.

[13] Nielsen AA, Voigt CA (2014). Multi-input CRISPR/Cas genetic circuits that interface host regulatory networks. Mol Syst Biol.

[14] Eeckhaut T, Lakshmanan PS, Deryckere D, Van Bockstaele E, Van Huylenbroeck J (2013). Progress in plant protoplast research. Planta.

[15] Mikami M, Toki S, Endo M (2015). Comparison of CRISPR/Cas9 expression constructs for efficient targeted mutagenesis in rice. Plant Mol Biol.

[16] Patron NJ, Orzaez D, Marillonnet S, Warzecha H, Matthewman C, Youles M (2015). Standards for plant synthetic biology: a common syntax for exchange of DNA parts. New Phytol.

[17] Liu W, Stewart CN (2015). Plant synthetic biology. Trends Plant Sci.

[18] Sarrion-Perdigones A, Vazquez-Vilar M, Palaci J, Castelijns B, Forment J, Ziarsolo P (2013). GoldenBraid 2.0: a comprehensive DNA assembly framework for plant synthetic biology. Plant Physiol.

[19] Vazquez-Vilar M, Sarrion-Perdigones A, Ziarsolo P, Blanca J, Granell A, Orzaez D (2015). Software-assisted stacking of gene modules using GoldenBraid 2.0 DNA-assembly framework. Methods Mol Biol.

[20] Duportet X, Wroblewska L, Guye P, Li Y, Eyquem J, Rieders J (2014). A platform for rapid prototyping of synthetic gene networks in mammalian cells. Nucleic Acids Res.

[21] Guo Y, Dong J, Zhou T, Auxillos J, Li T, Zhang W (2015). YeastFab: the design and construction of standard biological parts for metabolic engineering in *Saccharomyces cerevisiae*. Nucleic Acids Res.

[22] Engler C, Gruetzner R, Kandzia R, Marillonnet S (2009). Golden gate shuffling: a one-pot DNA shuffling method based on type IIs restriction enzymes. PLoS ONE.

[23] Sarrion-Perdigones A, Falconi EE, Zandalinas SI, Juarez P, Fernandez-del-Carmen A, Granell A (2011). GoldenBraid: an iterative cloning system for standardized assembly of reusable genetic modules. PLoS ONE.

[24] Lei Y, Lu L, Liu HY, Li S, Xing F, Chen LL (2014). CRISPR-P: a web tool for synthetic single-guide RNA design of CRISPR-system in plants. Mol Plant.

[25] Mali P, Yang L, Esvelt KM, Aach J, Guell M, DiCarlo JE (2013). RNA-guided human genome engineering via Cas9. Science.

[26] Li JF, Norville JE, Aach J, McCormack M, Zhang D, Bush J (2013). Multiplex and homologous recombination-mediated genome editing in *Arabidopsis* and *Nicotiana benthamiana* using guide RNA and Cas9. Nat Biotechnol.

[27] Bikard D, Jiang W, Samai P, Hochschild A, Zhang F, Marraffini LA (2013). Programmable repression and activation of bacterial gene expression using an engineered CRISPR-Cas system. Nucleic Acids Res.

[28] Xie K, Minkenberg B, Yang Y (2015). Boosting CRISPR/Cas9 multiplex editing capability with the endogenous tRNA-processing system. Proc Natl Acad Sci USA.

[29] Weber E, Engler C, Gruetzner R, Werner S, Marillonnet S (2011). A modular cloning system for standardized assembly of multigene constructs. PLoS ONE.

[30] Sakuma T, Nishikawa A, Kume S, Chayama K, Yamamoto T (2014). Multiplex genome engineering in human cells using all-in-one CRISPR/Cas9 vector system. Sci Rep.

[31] Ma X, Zhang Q, Zhu Q, Liu W, Chen Y, Qiu R (2015). A robust CRISPR/Cas9 system for convenient, high-efficiency multiplex genome editing in monocot and dicot plants. Mol Plant.

[32] Lowder LG, Zhang D, Baltes NJ, Paul JW, Tang X, Zheng X (2015). A CRISPR/Cas9 toolbox for multiplexed plant genome editing and transcriptional regulation. Plant Physiol.

[33] Nekrasov V, Staskawicz B, Weigel D, Jones JD, Kamoun S (2013). Targeted mutagenesis in the model plant *Nicotiana benthamiana* using Cas9 RNA-guided endonuclease. Nat Biotechnol.

[34] Upadhyay SK, Kumar J, Alok A, Tuli R (2013). RNA-guided genome editing for target gene mutations in wheat. G3.

[35] Senis E, Fatouros C, Grosse S, Wiedtke E, Niopek D, Mueller AK (2014). CRISPR/Cas9-mediated genome engineering: an adeno-associated viral (AAV) vector toolbox. Biotechnol J.

[36] Port F, Chen HM, Lee T, Bullock SL (2014). Optimized CRISPR/Cas tools for efficient germline and somatic genome engineering in Drosophila. Proc Natl Acad Sci USA.

[37] Xing HL, Dong L, Wang ZP, Zhang HY, Han CY, Liu B (2014). A CRISPR/Cas9 toolkit for multiplex genome editing in plants. BMC Plant Biol.

