# Supplementary material for: A modular toolbox for gRNA–Cas9 genome engineering in plants based on the GoldenBraid standard
Source: Plant Methods. 2016 Feb 1;12:10. doi: 10.1186/s13007-016-0101-2 (PMC4736081; doi:10.1186/s13007-016-0101-2)
Supplement: Supplementary file 1 — 10.1186/s13007-016-0101-2 Cloning efficiency of representative Level ≥1 GBelements. Figure S2. Schema of the dead Cas9 transcriptional units tested on the repression and activation experiments. [file 13007_2016_101_MOESM1_ESM.pdf]

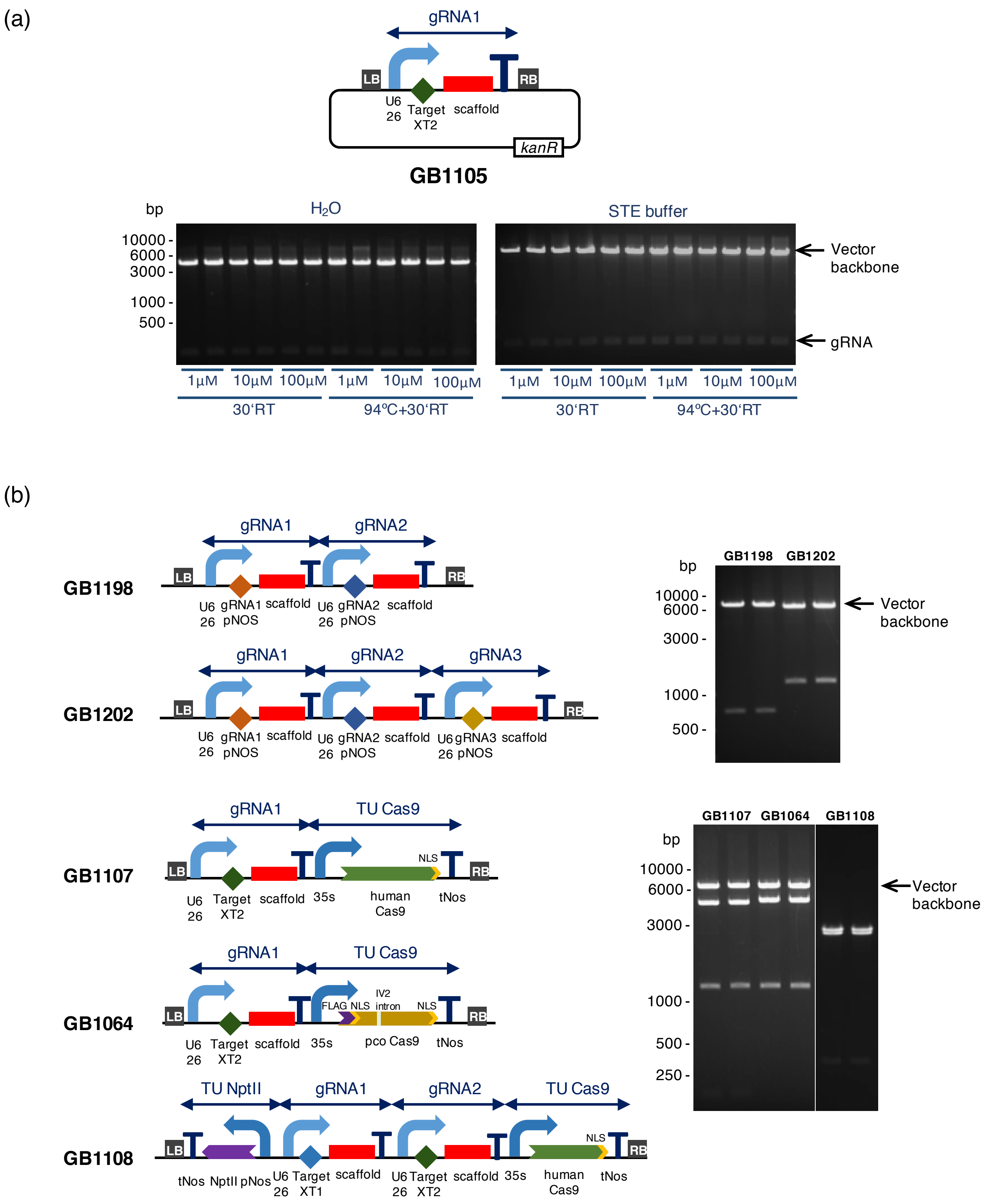

Figure S1. Cloning efficiency of representative Level≥1 GBelements. (a) Restriction analysis of 24 plasmids obtained from randomly selected colonies to test the accuracy of gRNA assemblies under different conditions (HindIII expected bands: 6345-374). (b) Restriction analysis of GB binary reactions combining gRNAs with each other and/or with the Cas9-encoding TU. All tested plasmids resulted in the expected restriction patterns. GB1198 BamHI expected bands: (6674-728); GB1202 EcoRI expected bands: (6345-1242); GB1107 EcoRV expected bands: (6674-4620-1236-194); GB1064 EcoRV expected bands: (6674-5117-1236); GB1108 EcoRI expected bands: (6987-6345-828).

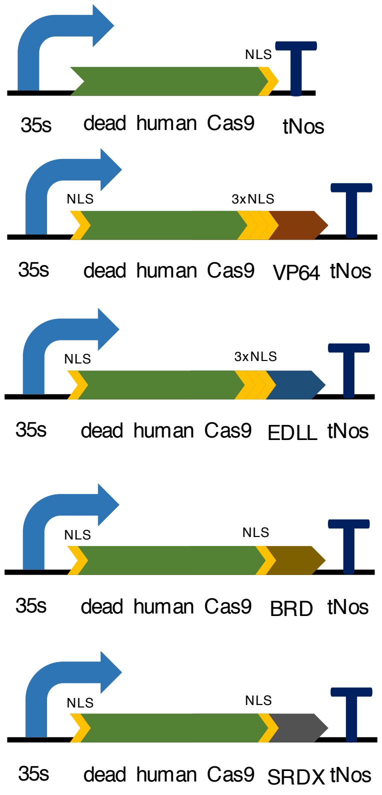

Figure S2. Schema of the architecture of the dead Cas9 transcriptional units tested on the repression and activation experiments.
