# Supplementary material for: A modular toolbox for gRNA–Cas9 genome engineering in plants based on the GoldenBraid standard
Source: Plant Methods. 2016 Feb 1;12:10. doi: 10.1186/s13007-016-0101-2 (PMC4736081; doi:10.1186/s13007-016-0101-2)
Supplement: Supplementary file 2 — 10.1186/s13007-016-0101-2 Primers used for the amplification of the N. benthamiana xylosyltransferases XT1 (Niben101Scf04205Ctg025) and XT2 (Niben101Scf04551Ctg021) regions. Table S2. List of forward and reverse primers used to construct the targets. Table S3. List of GBelements generated in this work. [file 13007_2016_101_MOESM2_ESM.pdf]

**Table S1.** Primers used for the amplification of the *N.benthamiana* xylosyltransferases XT1 (Niben101Scf04205Ctg025) and XT2 (Niben101Scf04551Ctg021) regions.

| | |
|---|---|
| XT1_F | 5´-AACCACTTTTCCTCGTCGGAAA-3’ |
| XT1_R | 5´-TAACTATTCAACTAAAGCTTCAAACAG-3’ |
| XT2_F | 5’-AACCACTTTTCCTTGTCGGAAA-3’ |
| XT2_R | 5’-GGAATGAAATTAACCACTTCAGG-3’ |
| XT12BsaI_F | 5’-GCGGGTCTCAGGAGCCCTAATGTTGCTTGGAGATC-3’ |
| XT12BsaI_R | 5´-GCGGGTCTCAAGCGCCGTCTAAGGTTCAATTTGAGTAGC-3’ |

**Table S2.** List of forward and reverse primers used to construct the targets.

| | |
|---|---|
| gRNA XT1_F | 5′-ATTGAAAACACCGTCTTCGGAGA-3’ |
| gRNA XT1_R | 5′-AAACTCTCCGAAGACGGTGTTTT-3’ |
| gRNA XT2_F | 5′-ATTGAAAATTGGGAAAAAACTAG-3’ |
| gRNA XT2_R | 5′-AAACCTAGTTTTTTCCCAATTTT-3’ |
| gRNA1 pNOS_F | 5’-ATTGAGACTCTAATTGGATACCG-3’ |
| gRNA1 pNOS_R | 5’-AAACCGGTATCCAATTAGAGTCT-3’ |
| gRNA2 pNOS_F | 5’-ATTGACGTTCCATAAATTCCCCT-3’ |
| gRNA2 pNOS_R | 5’-AAACAGGGGAATTTATGGAACGT-3’ |
| gRNA3 pNOS_F | 5’-ATTGACTTTTGAACGCGCAATAA-3’ |
| gRNA3 pNOS_R | 5’-AAACTTATTGCGCGTTCAAAAGT-3’ |
| gRNA4 pNOS_F | 5’-ATTGCCACTGAGCCGCGGGTTTC-3’ |
| gRNA4 pNOS_R | 5’-AAACGAAACCCGCGGCTCAGTGG-3’ |
| gRNA5 pNOS_F | 5’-ATTGGGACAAGCCGTTTTACGTT-3’ |
| gRNA5 pNOS_R | 5’-AAACAACGTAAAACGGCTTGTCC-3’ |
| gRNA FT5447tRNA_F* | 5’-GTGCACCCAAAAGAAATGGTCCAAT-3’ |
| gRNA FT5447tRNA_R* | 5’-AAACATTGGACCATTTCTTTTGGGT-3’ |
| gRNA FT1272tRNA_F* | 5’-GTGCACCAATAAGCAATGGCGCAAT-3’ |
| gRNA FT1272tRNA_R* | 5’-AAACATTGCGCCATTGCTTATTGGT-3’ |
| gRNA FT2631tRNA_F* | 5’-GTGCACCGATAAACAATGGCGCAAT-3’ |
| gRNA FT2631tRNA_R* | 5’-AAACATTGCGCCATTGTTTATCGGT-3’ |
| gRNA XT4205tRNA_F* | 5’-GTGCAGAAAACACCGTCTTCGGAGA-3’ |
| gRNA XT4205tRNA_R* | 5’-AAACTCTCCGAAGACGGTGTTTTCT-3’ |
| gRNA XT4551tRNA_F* | 5’-GTGCAGAAAATTGGGAAAAAACTAG-3’ |
| gRNA XT4551tRNA_R* | 5’-AAACCTAGTTTTTTCCCAATTTTCT-3’ |

*Primers used on the assembly of GBoligomers on level 0.

**Table S3.** List of GBelements generated in this work.

**Level -1 GBparts**

| GBdatabase ID | Name |
|---|---|
| GB1205 | tRNA-gRNA position [D1_2] |
| GB1206 | tRNA-gRNA position [2_n-1] |
| GB1207 | tRNA-gRNA position [n] |
| GB1208 | tRNA-gRNA position [D1_n-1] |
| GB1209 | tRNA-gRNA position [M1_2] |
| GB1210 | tRNA-gRNA position [M1_n-1] |

**Level 0 GBparts**

| GBdatabase ID | Name | Category |
|---|---|---|
| GB0273 | ppcoCas9 | B3-B4-B5 |
| GB0575 | phCas9 | B3-B4-B5 |
| GB0645 | psgRNA | B6b-C1 |
| GB1001 | pAtU6-26 | A1-A2-A3-B1-B2c |
| GB1041 | pdCas9 | B3-B4-B5 |
| GB1079 | pdCas9 | B3-B4 |
| GB1175 | pNLS-BRD | B5 |
| GB1184 | pOsU3 | A1-A2-A3-B1-B2d |
| GB1185 | pNLS-SRDX | B5 |
| GB1186 | p3xNLS-VP64 | B5 |
| GB1187 | p3xNLS-EDLL | B5 |
| GB1204 | pAtU6-1 | A1-A2-A3-B1-B2c |
| GB1211 | ptRNA-target5447_5494_17626FT-gRNA[D1_2] | Other |
| GB1212 | ptRNA-target1272FT-gRNA[2_n-1] | Other |
| GB1213 | ptRNA-target2631FT-gRNA[n] | Other |
| GB1214 | ptRNA-target4205XT-gRNA[D1_n-1] | Other |
| GB1215 | ptRNA-target4551XT-gRNA[n] | Other |

**Level 1 GB TUs**

| GBdatabase ID | Name | % accuracy* |
|---|---|---|
| GB0639 | pEGB2α2 35s:hCas9:tNOS | 100% |
| GB0576 | pEGB3α2 35s:pcoCas9:tNOS | 50% |
| GB1104 | pEGB3α1 U626:gRNAXT4551:sgRNA | 100% |
| GB1105 | pEGB3α2 U626:gRNAXT4205:sgRNA | 100% |
| GB1172 | pEGB3α2 35s:hCas9:BRD:tNOS | 100% |
| GB1177 | pEGB3α1 U626:gRNA1pNOS:sgRNA | 100% |
| GB1178 | pEGB3α2 U626:gRNA1pNOS:sgRNA | 100% |
| GB1179 | pEGB3α1 U626:gRNA2pNOS:sgRNA | 100% |
| GB1180 | pEGB3α1 U626:gRNA4pNOS:sgRNA | 100% |
| GB1188 | pEGB3α2 35s:hCas9:SRDX:tNOS | 100% |
| GB1189 | pEGB3α2 35s:hCas9:VP64:tNOS | 100% |
| GB1190 | pEGB3α2 35s:hCas9:EDLL:tNOS | 100% |
| GB1191 | pEGB3α2 35s:dCas9:tNOS | 100% |
| GB1192 | pEGB3α1 U626:gRNA3pNOS:sgRNA | 100% |
| GB1193 | pEGB3α1 U626:gRNA5pNOS:sgRNA | 100% |
| GB1194 | pEGB3α2 U626:gRNA3pNOS:sgRNA | 100% |
| GB1195 | pEGB3α2 U626:gRNA5pNOS:sgRNA | 100% |
| GB1196 | pEGB3α2 U626:gRNA2pNOS:sgRNA | 75% |
| GB1197 | pEGB3α2 U626:gRNA4pNOS:sgRNA | 100% |
| GB1221 | pEGB3α1 U626:gRNA1pDFR:sgRNA | 100% |

| Level >1 GB Modules | | |
|---|---|---|
| **GBdatabase ID** | **Name** | |
| GB1064 | pEGB3Ω2 U6-26:target4551XT:sgRNA-35s:pcoCas9:tNOS | 75% |
| GB1106 | pEGB3Ω1 tNOS:nptII:pNOS-U626:target4205XT:sgRNA | 100% |
| GB1107 | pEGB3Ω2 U6-26:target4551XT:sgRNA-35s:hCas9:tNOS | 100% |
| GB1108 | pEGB 3α1 tNOS:nptII:pNOS-U626:target4205XT:sgRNA-U626:target4551XT:sgRNA-35s:hCas9:tNOS | 100% |
| GB1116 | pEGB3α1 pNOS:Luciferase:tNOS-SF-35S:Renilla:tNOS-35S:P19:tNOS-SF | 100% |
| GB1198 | pEGB3Ω1 U626:gRNA1pNOS:sgRNA-U626:gRNA2pNOS:sgRNA | 100% |
| GB1199 | pEGB3Ω1 U626:gRNA1pNOS:sgRNA-U626:gRNA4pNOS:sgRNA | 100% |
| GB1200 | pEGB3Ω1 U626:gRNA2pNOS:sgRNA-U626:gRNA4pNOS:sgRNA | 100% |
| GB1201 | pEGB3Ω2 U626:gRNA4pNOS:sgRNA-SF | 100% |
| GB1202 | pEGB3α1 U626:gRNA1pNOS:sgRNA-U626:gRNA2pNOS:sgRNA-U626:gRNA4pNOS:sgRNA | 100% |
| GB1216 | pEGB3α1 U626:tRNA-target1FT-gRNA:tRNA-target2FT:gRNA:tRNA-target3FT-gRNA | 100% |
| GB1217 | pEGB3α2 U626:tRNA-target1XT-gRNA:tRNA-target2XT:gRNA | 100% |
| GB1218 | pEGB3Ω1 U626:tRNA-gRNA(x3withFTtargets)-U626:tRNA-gRNA(x2withXTtargets) | 100% |
| GB1219 | pEGB3Ω1 U626:gRNA3pNOS:sgRNA-U626:gRNA5pNOS:sgRNA | 100% |
| GB1220 | pEGB3α1 U626:gRNA3pNOS:sgRNA-U626:gRNA5pNOS:sgRNA-U626:gRNA4pNOS:sgRNA | 100% |
| GB1222 | pEGB3α1 U626:tRNA-gRNA(x3withFTtargets)-U626:tRNA-gRNA(x2withXTtargets)-35s:hCas9:tNOS | 100% |

* Cloning accuracy expressed as the % of colonies showing the correct restriction pattern (number of colonies assayed 2-4).
